# CobB regulates *Escherichia coli* chemotaxis by deacetylating the response regulator CheY

**DOI:** 10.1111/j.1365-2958.2010.07125.x

**Published:** 2010-04-13

**Authors:** Ru Li, Jing Gu, Yuan-Yuan Chen, Chuan-Le Xiao, Li-Wei Wang, Zhi-Ping Zhang, Li-Jun Bi, Hong-Ping Wei, Xu-De Wang, Jiao-Yu Deng, Xian-En Zhang

**Affiliations:** 1 State Key Laboratory of Virology, Wuhan Institute of Virology, Chinese Academy of SciencesWuhan 430071, China; 2 Graduate School, Chinese Academy of ScienceBeijing 100039, China; 3 National Laboratory of Biomacromolecules, Institute of Biophysics, Chinese Academy of SciencesBeijing 100101, China; 4 Institute of Life and Health Engineering, Jinan UniversityGuangzhou 510632, China; 5 National Engineering and Research Center for Genetic Medicine, Jinan UniversityGuangzhou 510632, China

## Abstract

The silent information regulator (Sir2) family proteins are NAD^+^-dependent deacetylases. Although a few substrates have been identified, functions of the bacteria Sir2-like protein (CobB) still remain unclear. Here the role of CobB on *Escherichia coli* chemotaxis was investigated. We used Western blotting and mass spectrometry to show that the response regulator CheY is a substrate of CobB. Surface plasmon resonance (SPR) indicated that acetylation affects the interaction between CheY and the flagellar switch protein FliM. The presence of intact flagella in knockout strains Δ*cobB*, Δ*acs*, Δ(*cobB*) Δ(*acs*), Δ(*cheA*) Δ(*cheZ*), Δ(*cheA*) Δ(*cheZ*) Δ(*cobB*) and Δ(*cheA*) Δ(*cheZ*) Δ(*acs*) was confirmed by electron microscopy. Genetic analysis of these knockout strains showed that: (i) the Δ*cobB* mutant exhibited reduced responses to chemotactic stimuli in chemotactic assays, whereas the Δ*acs* mutant was indistinguishable from the parental strain, (ii) CheY from the Δ*cobB* mutant showed a higher level of acetylation, indicating that CobB can mediate the deacetylation of CheY *in vivo*, and (iii) deletion of *cobB* reversed the phenotype of Δ(*cheA*) Δ(*cheZ*). Our findings suggest that CobB regulates *E. coli* chemotaxis by deacetylating CheY. Thus a new function of bacterial *cobB* was identified and also new insights of regulation of bacterial chemotaxis were provided.

## Introduction

Members of the Sir2 family of proteins are nicotinamide adenine dinucleotide (NAD^+^)-dependent deacetylase enzymes that modulate gene silencing (Rusche *et al*., 2003), cell cycle regulation (Dryden *et al*., 2003), fatty acid metabolism (Starai *et al*., 2002), lifespan extension (Rogina and Helfand, 2004) and apoptosis (Langley *et al*., 2002). Many organisms contain multiple orthologues of the Sir2 family proteins, and thus it is expected that other substrates under the control of Sir2 proteins will be revealed (Starai *et al*., 2004). In *Salmonella enterica* it has been reported that acetylation blocks the adenylating activity of the enzyme acetyl-CoA synthetase (Acs) and activation of the acetylated enzyme requires the activity of the Sir2-like protein CobB (Starai *et al*., 2002). When the concentration of acetate in the environment is low, Acs is responsible for conversion of acetate to acetyl-CoA (AcCoA) (Starai *et al*., 2003). However, the physiological roles of CobB in bacteria still remain largely unknown.

Chemotaxis is a mechanism by which bacteria respond to changes in the chemical compositions of their environment, approaching attractants and avoiding repellents. The chemotactic response of bacteria such as *Escherichia coli* is accomplished by signal transmission between two supramolecular complexes: the receptor complexes and the flagellar–motor complexes. The response regulator of bacterial chemotaxis, CheY, shuttles back and forth between the complexes and transduces the signal from the receptors to the flagella (Silversmith and Bourret, 1999; Sourjik, 2004). CheY can be modulated by two covalent modifications: phosphorylation and acetylation (Baker *et al*., 2006). CheY is phosphorylated by phosphoryl groups either by its associated autophosphorylating protein kinase, CheA, or by small phosphodonor molecules such as acetyl phosphate (acetyl-P). In fact, the contribution of acetyl-P is minimal because the phosphotransfer rate from CheA to CheY is orders of magnitude faster than the rate from acetyl-P to CheY (Wolfe, 2005). The phosphorylated form, CheY-P, binds to the switch protein FliM much better than does the non-phosphorylated form at the base of the flagellar motor, which then increases the probability of shifting the direction of flagellar rotation from the default direction, counterclockwise (CCW), to clockwise (CW) (Welch *et al*., 1993). CheY-P dephosphorylates spontaneously, an activity enhanced by another chemotaxis protein, CheZ (Zhao *et al*., 2002). This dephosphorylation reduces the binding of CheY to the switch (Bren *et al*., 1996).

The acetylation of CheY is carried out by acetyl-CoA synthetase (Acs, with acetate as the acetyl donor) (Barak *et al*., 1992) or by autoacetylation (with AcCoA as the acetyl donor) (Barak *et al*., 2006). Recently, the acetylation of CheY has been detected *in vivo*, and it was found to result mainly from CheY autoacetylation (Yan *et al*., 2008). The acetylation sites are six lysine residues – lysines 91, 92, 109, 119, 122 and 126, all clustered at the C-terminus of the protein and localized on the surface that binds FliM, CheZ and CheA (Barak *et al*., 2004). Although acetylation, like phosphorylation, appears to influence chemotaxis of *E. coli* (Barak *et al*., 1998; Ramakrishnan *et al*., 1998; Barak and Eisenbach, 2001), the role of CheY acetylation in bacterial chemotaxis still remains obscure.

Although CheY was known to undergo acetylation, enzymes responsible for its deacetylation were not fully investigated. Since CobB was shown to be a protein deacetylase, it is possible that CheY might be one of its substrates and so the correlation between CobB and chemotaxis of *E. coli* was carefully investigated. We speculated that CobB could catalyse CheY deacetylation and hence regulate bacterial chemotaxis. Here, we addressed this speculation very carefully by using biochemical and genetic analysis. To our knowledge, this is the first time to show that CobB regulates *E. coli* chemotaxis by deacetylating CheY. This broadens our understandings of the physiological roles of Sir2 family protein in bacteria and also provides new insights into the regulation of bacterial chemotaxis.

## Results

### CobB deacetylates Acs and CheY *in vitro*

To test whether CobB could deacetylate Acs in *E. coli*, acetylated Acs (AcAcs) was incubated with CobB in the presence of NAD^+^. We performed Western blotting analysis to test the acetylation level of Acs using an anti-acetyl-lysine antibody. The result demonstrated that CobB markedly reduces the acetylation level of AcAcs, and that this effect can be alleviated in the presence of the Sir2 inhibitor nicotinamide (NAM) (Fig. S1).

**Fig. 1:**
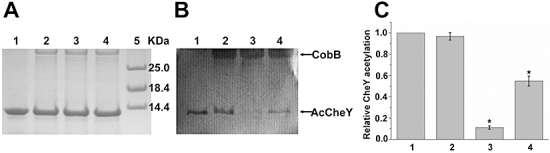
CobB deacetylates AcCheY *in vitro*. The acetylation levels of all proteins were determined by Western blotting using a specific anti-acetyl-lysine antibody. Experiments were replicated three times, and representative results are shown. A and B. (A) SDS-PAGE of samples. (B) Western blot of the gel in (A). Lane 1, AcCheY + NAD^+^; lane 2, AcCheY + CobB; lane 3, AcCheY + CobB + NAD^+^; lane 4, AcCheY + CobB + NAD^+^ + NAM; lane 5, marker. The concentration of AcCheY and CobB was 15 µM and 3 µM respectively. The reaction was carried out for 6 h at 25°C. C. Average levels of CheY acetylation, quantified from the Western blots of three experiments (mean ± SD) using AlphaView image analysis software and normalized relative to the value obtained in absence of CobB (lane 1). An asterisk indicates a statistically significant difference from lane 1 (*P* < 0.01; anova analysis).

To provide primary evidence that CheY is the substrate of CobB, acetylated CheY (AcCheY) was deacetylated and protein acetylation level was analysed with an anti-acetyl-lysine antibody. As shown in Fig. 1, the acetylation level of AcCheY was not affected in the absence of CobB or NAD^+^ (lanes 1, 2). In contrast, the acetylation level of AcCheY decreased greatly in the presence of CobB and NAD^+^ (lane 3). NAM reduced the deacetylation of AcCheY by CobB (lane 4). Additionally, the acetylation level of CobB itself is relatively high, consistent with a recent report that human Sirt2, a member of the Sir2 family, can be acetylated by p300, a protein with protein acetyltransferase activity (Han *et al*., 2008). These results clearly demonstrate that in *E. coli*, CobB can deacetylate CheY *in vitro*.

**Fig. 2:**
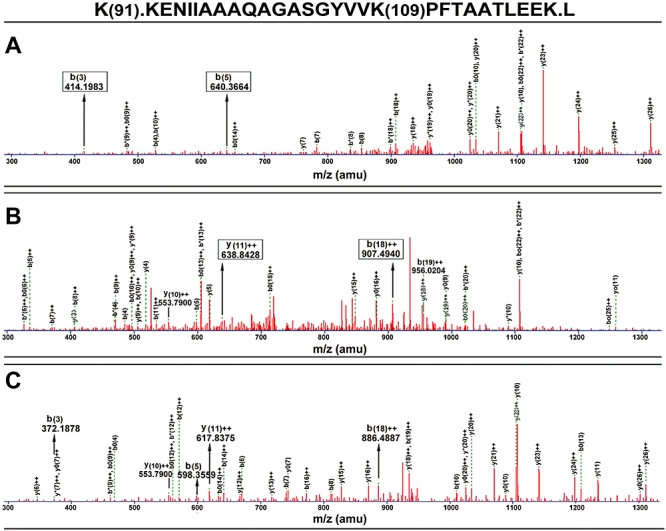
LC-MS/MS analysis confirms that lysine 91 and 109 are acetylated in AcCheY, and can be deacetylated by CobB. A. Tryptic digestion of AcCheY with Lys91 acetylated. The boxed b(3) and b(5) ions have a mass of 42 Da greater than the corresponding ions in the CobB-treated sample. B. Tryptic digestion of AcCheY with Lys109 acetylated. The boxed b(18)^++^ and y(11)^++^ ions have a mass of 21 Da greater than the corresponding ions in the CobB-treated sample. C. Tryptic digestion of D-CheY.

To identify the site(s) of acetylation and deacetylation, AcCheY and D-CheY (deacetylated AcCheY by CobB) were prepared, enzymatically digested and analysed by LC-MS in a LTQ-Orbitrap mass spectrometer. Lysine-acetylated peptide can be identified as they have a mass increment of 42 Da (Larsen *et al*., 2006). The acetylated peptide in AcCheY was shown to be K(91).KENIIAAAQAGASGYVVK(109)PFTAATLEEK.L, and had a molecular mass of 2918.55, +42 Da heavier than the equivalent peptide in D-CheY (2876.53). The fragment ion signals reflect the amino acid sequence as read from either the N-terminal (b-ion series) or the C-terminal (y-ion series) direction. As shown in Fig. 2A, the boxed b(3) and b(5) have a mass of 42 Da greater than the corresponding ions in the CobB-treated sample (Fig. 2C). This indicates that the N-terminal residue lysine 91 is acetylated in AcCheY. The boxed b(18)^++^ and y(11)^++^ ions in Fig. 2B have a mass of 21 Da greater than the corresponding ions in the Fig. 2C. Subsequent ions in the series b(19–27)^++^ and y(12–27)^++^ all showed a mass increased by 21 Da in the acetylated sample. This indicates the presence of an acetyl group on lysine 109 in AcCheY. More detailed data are available in Tables S1 and S2. Thus, these mass spectrometry results reveal that the acetylated residues in CheY are lysine 91 and 109, and that CobB can catalyse CheY deacetylation at the same sites.

### Influence of acetylation on the interaction between CheY and FliM

**Fig. 3:**
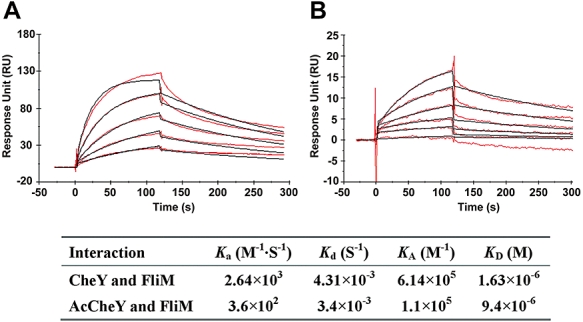
SPR analysis of CheY and AcCheY binding to FliM. Approximately 1120 RU FliM was immobilized on CM5 sensor surface using amine coupling method. This experiment was replicated at least three times. A. Responses of CheY at the concentrations 1–16 µM (from lower to upper). B. Responses of AcCheY at the concentrations 1–32 µM (from lower to upper). The red lines represent protein injections at the indicated concentration. The black lines represent the global fit of the entire data set to 1:1 Langmuir binding model. The inserted table lists the kinetic constants derived from the sensorgrams.

To investigate whether acetylation of CheY affects the interaction between CheY and FliM, surface plasmon resonance (SPR), a sensitive technology to measure the molecular interactions, was employed to study interaction between FliM and CheY. The SPR 3000 system contains a dual-channel measuring cell. The working channel is linked to a sensor chip while the reference channel is linked to the same chip without the immobilized sensor element protein His-FliM. As shown in Fig. 3, interactions between His-FliM and CheY-His (or AcCheY-His) were detected. The response unit (RU) values were proportional to sample concentrations. At the same sample concentration, AcCheY-His exhibited a significantly lower binding signal with His-FliM, compared with CheY-His. The *K*_D_ values of interactions between His-FliM and AcCheY-His, and between His-FliM and CheY-His are 9.4 and 1.63 µM respectively. These results therefore suggest that acetylation of CheY reduces its binding affinity to FliM.

### Chemotactic behaviour of *ΔcobB*, *Δacs* and *Δ*(*cobB*) *Δ*(*acs*) mutants

To investigate whether CobB can regulate chemotaxis of *E. coli*, a Δ*cobB* mutant (RL001) was constructed and its phenotype was tested. In 2003, Starai *et al*. reported that *cobB* mutants have trouble growing on low concentrations of acetate because Acs remains acetylated, which means that Acs remains constitutively off (Starai *et al*., 2003). Since growth on low concentration of acetate requires Acs (Kumari *et al*., 1995), *cobB* mutants grow poorly on low acetate. In our study, 10 mM acetate was used as the lowest concentration to test the ability of RL001 to grow on acetate. Figure S2 shows that the Δ*cobB* mutant (RL001) grew poorly on low levels of acetate. Thus, this result confirms that the Δ*cobB* mutant behaves as expected.

In order to test whether flagellum synthesis or function was affected by deletion of *cobB*, RL001 was observed under the electron microscope. As shown in Fig. S3, RL001 exhibited normal shape and similar numbers of flagella as W3110. Since flagellum assembly in the Δ*cobB* mutant is normal, we speculated that CobB might affect chemotaxis. To this end, the effect of RL001 on chemotaxis was tested by several distinct chemotaxis assays. Furthermore, in order to rule out the possibility that the effect of CobB on chemotaxis may be caused by its ability to deacetylate Acs, the chemotactic responses of the Δ*acs* mutant (RL002) and Δ(*cobB*) Δ(*acs*) (RL0012) mutant were also examined.

**Fig. 4:**
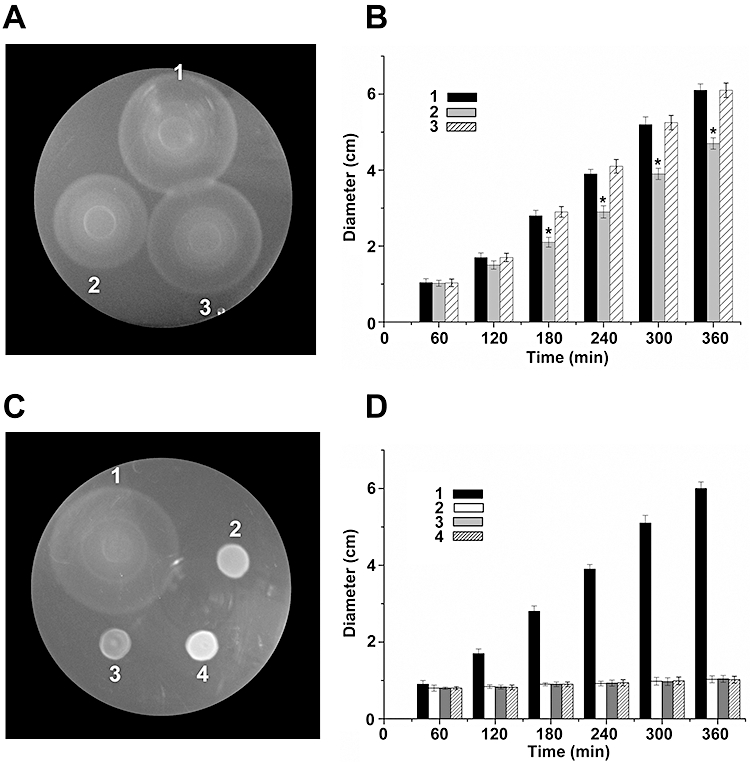
Swarm assays on semi-solid TB plates. Cells were inoculated near the centre of tryptone swarm plates containing 0.2% agar and incubated at 35°C. Experiments were replicated four times, and representative results are shown. A. Swarm ring formation by: (1) wild-type strain W3110, (2) W3110 Δ*cobB* mutant and (3) W3110 Δ*acs* mutant. Photographs were taken after 3 h. B. Displacement (diameter) of the outermost edge of swarms of strains used in (A). Data are means ± SD from four independent experiments. An asterisk indicates a statistically significant difference from W3110 (*P* < 0.05; anova analysis). C. Swarm ring formation by: (1) wild-type strain W3110, (2) W3110 Δ(*cheA*) Δ(*cheZ*) mutant, (3) W3110 Δ(*cheA*) Δ(*cheZ*) Δ(*cobB*) mutant and (4) W3110 Δ(*cheA*) Δ(*cheZ*) Δ(*acs*) mutant. Photographs were taken after 3.5 h. D. Displacement (diameter) of the outermost edge of swarms of strains used in (C). Data are means ± SD from four independent experiments.

In the swarm assay, bacteria migrate outward from the site of inoculation; migration on semi-solid motility plates depends on: the ability to transport and metabolize serine, aspartate and threonine, the ability to assemble flagella, the ability to rotate those flagella, the ability to sense these attractants (serine, aspartate and threonine) gradients (Wolfe and Berg, 1989). On tryptone broth semi-solid agar, the parental strain W3110 formed at least two rings, as did the Δ*cobB* mutant (Fig. 4A). However, the migration rate of the mutant was slower than that of the parental strain. In contrast, the Δ*acs* mutant was indistinguishable from the W3110 parental strain (Fig. 4B). To confirm that the similarity between them was not strain dependent, strains AJW613 and its Δ*acs* mutant derivative AJW803 were also examined and similar results were observed (Fig. S4). In addition, the Δ(*cobB*) Δ(*acs*) mutant showed similar migration rates with the Δ*cobB* mutant (data not shown).

**Fig. 5:**
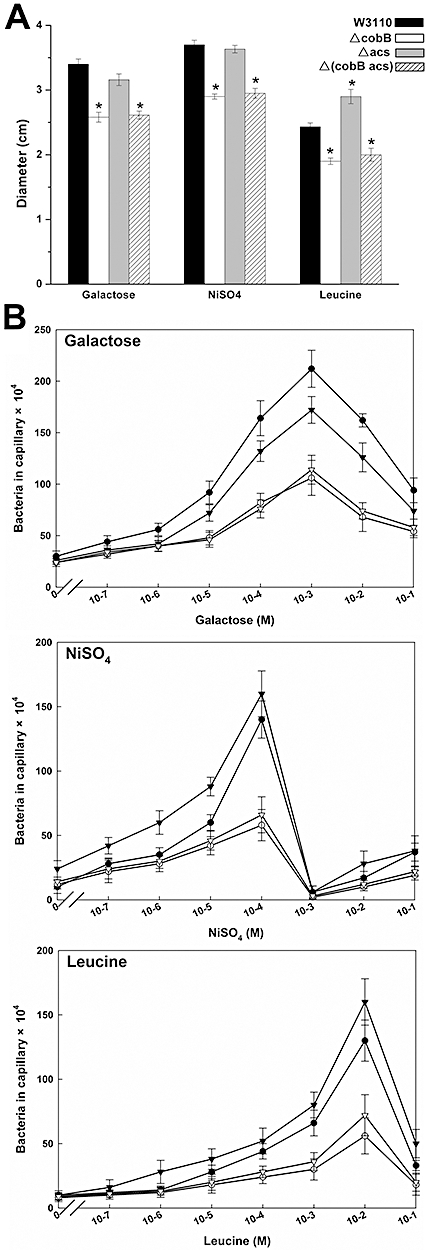
Chemotactic responses of W3110, Δ*cobB* mutant, Δ*acs* mutant and Δ(*cobB*) Δ(*acs*) mutant. A. Plug assays and drop assays. The concentrations of galactose plug, NiSO_4_ plug and leucine drop were 50 mM, 25 mM and 100 mM respectively. The medium was supplemented with 30 mM acetate. The results were quantified with a ruler. Data are means ± SD from four independent experiments. An asterisk indicates a statistically significant difference from W3110 (*P* < 0.05; anova analysis). B. Capillary assays. Assays were carried out at 35°C for 1 h. Only concentrations indicated by the symbols were tested. Data are means ± SD from three independent experiments. (▾) W3110; (○) W3110 Δ*cobB* mutant; (▾) W3110 Δ*acs* mutant; (▿) W3110 Δ(*cobB*) Δ(*acs*) mutant.

To determine what led to the reduced migration rate for the Δ*cobB* mutant, we used other assays (plug and capillary assays) that do not depend on the ability of the cells to establish their own gradient, that is the cells do not need to be able to transport and metabolize attractants or repellants. In the plug assay, chemotactically responsive bacteria in semi-solid agar accumulate near a plug containing an attractant or at a distance from a plug containing a repellent (Tso and Adler, 1974). When galactose (50 mM) was used as the attractant, results showed that strain W3110 accumulated normally near the galactose plug, while the accumulation of Δ*cobB* and Δ(*cobB*) Δ(*acs*) mutants near the plug was markedly reduced. However, only a very slight reduction was observed for the Δ*acs* mutant (Fig. S5, upper panel). Likewise, W3110 formed a bacteria-deficient zone around the plug containing the repellent NiSO_4_ (25 mM), but the repulsion of Δ*cobB* and Δ(*cobB*) Δ(*acs*) mutants was markedly reduced and the response of the Δ*acs* mutant was similar to W3110 (Fig. S5, middle panel). In a modified drop assay in which leucine (100 mM) was used, similar results were observed except that the repellent response of the Δ*acs* mutant seemed to be even more marked than that of W3110 (Fig. S5, lower panel). Quantified results are shown in Fig. 5A.

To further test the above results, capillary assays which do not depend on the ability of the cells to establish their own gradient were also performed. Concentration–response curves for various attractants and repellants are shown in Fig. 5B. The extent of accumulation of W3110 in the capillary increased with the concentration of the galactose attractant. In comparison, the extent of the accumulation of the Δ*cobB* and Δ(*cobB*) Δ(*acs*) mutants was reduced and the response was only about half of the wild-type strain. The response of the Δ*acs* mutant was greater than 80% of W3110. When capillaries containing only motility buffer were inserted into the suspensions of bacteria in which the repellent NiSO_4_ or leucine were present, typical concentration-dependent accumulations of wild-type cells were observed. Again, for each repellent, the response of Δ*cobB* and Δ(*cobB*) Δ(*acs*) appeared to be markedly reduced in comparison with W3110. In contrast, responses of the Δ*acs* mutant were similar with those of W3110.

These observations suggest that the deficiency in chemotaxis of the Δ*cobB* mutant is not due to a lack of Acs activity, and thus we conclude that CobB acts on chemotaxis by a mechanism that does not involve Acs. Since CheY is acetylated and CobB is a deacetylase, we speculated that CobB influences chemotaxis by altering the acetylation status of CheY.

### Analysis of the *in vivo* acetylation level of CheY

**Fig. 6:**
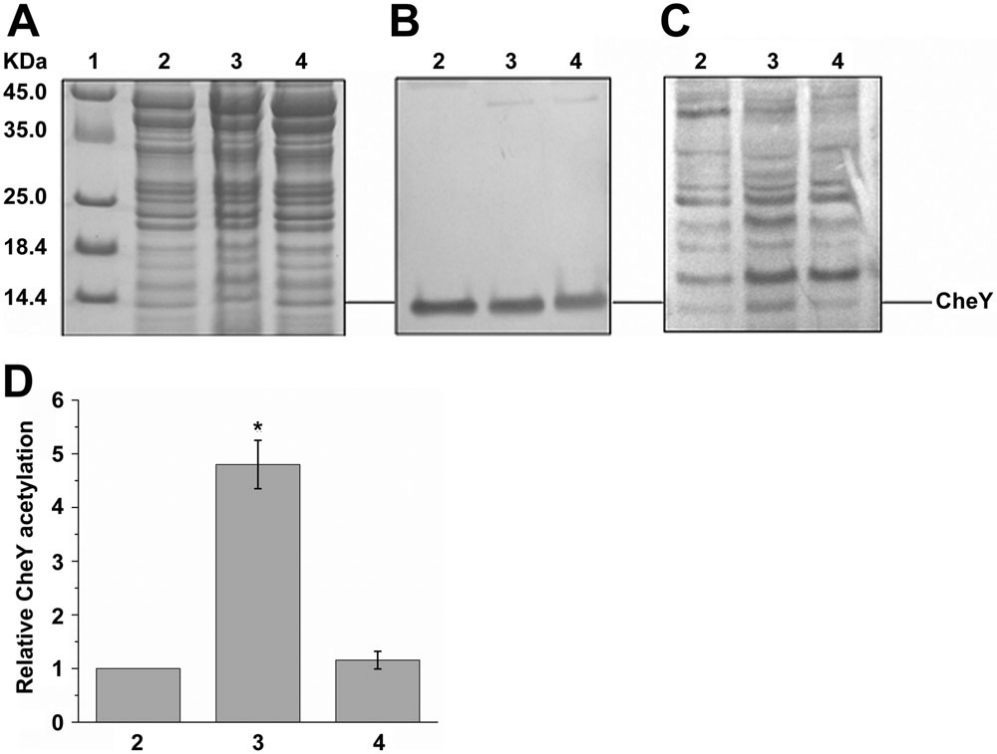
Western blotting analysis of CheY acetylation *in vivo*. The 30 µg protein lysate was resolved in 12–15% SDS-PAGE and analysed by Western blotting. Experiments were replicated three times, and representative results are shown. A–C. (A) SDS-PAGE of the extracts. (B) Western blot with a CheY antibody. (C) Western blot with an anti-acetyl-lysine antibody. Lane 1, marker; lane 2, W3110; lane 3, W3110 Δ*cobB* mutant; lane 4, W3110 Δ*acs* mutant. D. Average levels of CheY acetylation, quantified from the Western blots of three experiments (mean ± SD) using AlphaView image analysis software and normalized relative to the value obtained in W3110 (lane 2). An asterisk indicates a statistically significant difference from lane 2 (*P* < 0.01; anova analysis).

To provide more direct evidence for CheY deacetylation by CobB *in vivo*, CheY acetylation levels in strain W3110, and in the Δ*cobB* mutant (RL001) and Δ*acs* mutant (RL002) were analysed by Western blotting. As shown in Fig. 6A, the concentration of total proteins was similar in the three strains. Polyclonal anti-CheY antibodies were used to monitor CheY expression levels and the results (Fig. 6B) showed there was no difference in CheY expression level between these strains. However, Western blotting using an anti-acetyl-lysine antibody revealed that CheY acetylation level in the Δ*cobB* mutant was significantly higher than that of W3110, whereas only a slight difference was observed between W3110 and the Δ*acs* mutant (Fig. 6C and D). These data suggest that the absence of CobB results in an obvious increase of in the CheY acetylation level, and further indicates that CobB-mediated deacetylation has an effect on the CheY acetylation, not only *in vitro*, but also *in vivo*.

### Effects of *cobB* or *acs* deletion on the chemotactic behaviour of *Δ*(*cheA*) *Δ*(*cheZ*) mutant

In 2004, Barak and Eisenbach found that phosphorylation and acetylation of CheY affect with each other: CheA inhibits acetylation, whereas CheZ enhances it. Conversely, Acs enhances phosphorylation and acetate inhibits this enhancement (Barak and Eisenbach, 2004). Therefore, we chose to test the role of the *cobB* mutant in a *cheA cheZ* double mutant background. To achieve this, three mutants were constructed, including a *cheA cheZ* double mutant (RL003), a *cheA cheZ cobB* triple mutant (RL0031) and a *cheA cheZ acs* triple mutant (RL0032).

On a tryptone swarm plate, mutant RL003, RL0031 and RL0032 should not form chemotactic bands because they failed to respond to serine, aspartate and threonine chemical attractants. As shown in Fig. 4C and D, they did not form rings on 0.2% agar within 4 h at 35°C, unlike W3110. When the incubation time was prolonged, similar results were obtained.

**Fig. 7:**
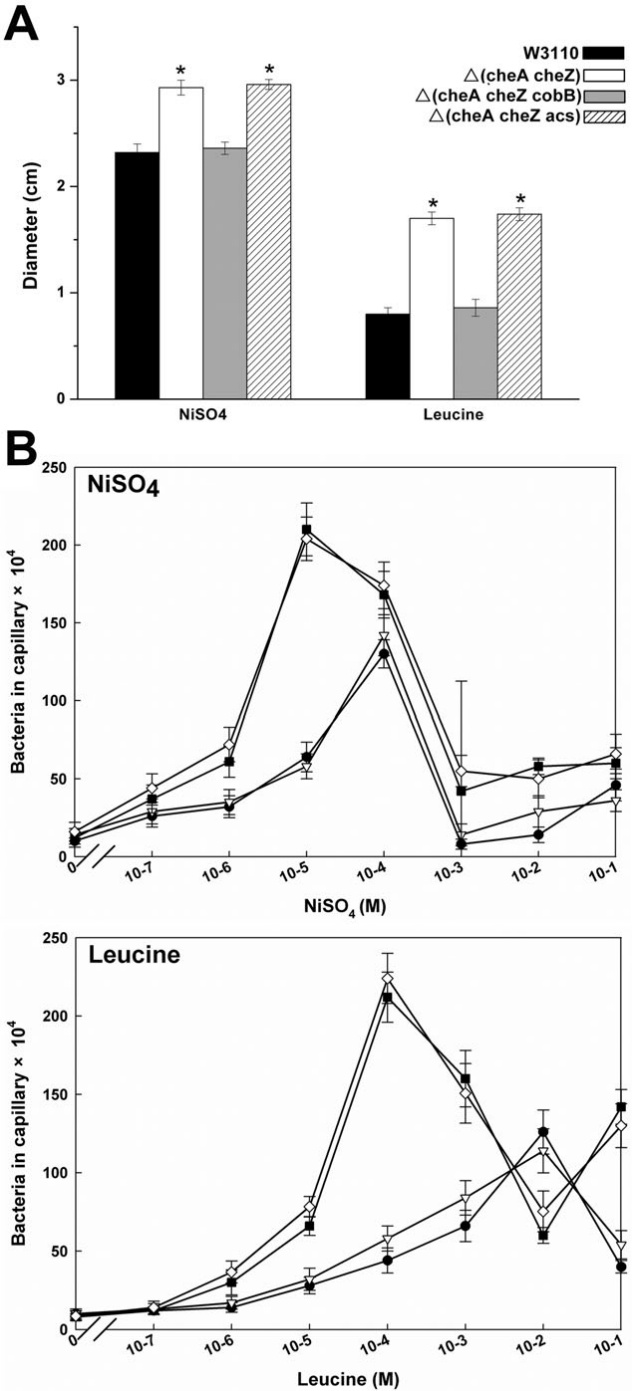
Chemotactic responses of W3110, Δ(*cheA*) Δ(*cheZ*), Δ(*cheA*) Δ(*cheZ*) Δ(*cobB*) and Δ(*cheA*) Δ(*cheZ*) Δ(*acs*). A. Plug assays and drop assays. The concentrations of NiSO_4_ plug, leucine drop and galactose plug were 25 mM, 100 mM and 50 mM respectively. The results were quantified with a ruler. Data are means ± SD from four independent experiments. An asterisk indicates a statistically significant difference from W3110 (*P* < 0.05; anova analysis). B. Capillary assays. Only the concentrations indicated by the symbols were tested. Data are means ± SD from three independent experiments. (▪) W3110; (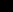) W3110 Δ(*cheA*) Δ(*cheZ*) mutant; (▿) W3110 Δ(*cheA*) Δ(*cheZ*) Δ(*cobB*) mutant; (◊) W3110 Δ(*cheA*) Δ(*cheZ*) Δ(*acs*) mutant.

Plug assay showed that all three strains formed a bacteria-free zone in the presence of a repellent-containing plug (NiSO_4_), and did not show any response to the attractant galactose (Fig. S6, upper and lower panels). Interestingly, the repulsion of the Δ(*cheA*) Δ(*cheZ*) mutant was greater than that of W3110, whereas Δ(*cheA*) Δ(*cheZ*) Δ(*cobB*) showed a similar degree of repulsion as W3110. However, the response of the Δ(*cheA*) Δ(*cheZ*) Δ(*acs*) mutant to the repellent was indistinguishable from the Δ(*cheA*) Δ(*cheZ*) mutant. Likewise, results obtained in the leucine-containing drop assay were similar to those of the plug assay (Fig. S6, middle panel). The plug assay was quantified as shown in Fig. 7A.

In the capillary assay, the responses of the Δ(*cheA*) Δ(*cheZ*) and Δ(*cheA*) Δ(*cheZ*) Δ(*acs*) mutants to the repellents (NiSO_4_ and leucine) were similar (Fig. 7B), which is consistent with our observations above. Furthermore, these two mutants were repelled by leucine into the capillaries, with a peak response at 10^−4^ M leucine. In contrast, the peak response of W3110 and the Δ(*cheA*) Δ(*cheZ*) Δ(*cobB*) mutant was about 10^−2^ M leucine (Fig. 7B). Thus, chemotactic responses of the Δ(*cheA*) Δ(*cheZ*) mutant and the Δ(*cheA*) Δ(*cheZ*) Δ(*acs*) mutant towards the repellents appear to be more sensitive and greater compared with those of W3110 and the Δ(*cheA*) Δ(*cheZ*) Δ(*cobB*) mutant.

Taken together, this study provides evidence that CobB does indeed participate in *E. coli* chemotaxis through deacetylation of CheY. Our results also confirm that the two covalent modifications of CheY, phosphorylation and acetylation, are linked *in vivo*.

## Discussion

In this study, we proposed that CobB regulates *E. coli* chemotaxis by deacetylating CheY. A new function of bacterial *cobB* gene was identified and new insights of regulation of bacterial chemotaxis were provided herein. The main findings are discussed below.

### AcCheY is a substrate of CobB

Several observations prompted us to speculate that AcCheY could be a substrate of CobB. CheY is known to undergo Acs-mediated acetylation or catalyse its own acetylation. We wondered whether CheY acetylation, like acetylation of histones and eukaryotic transcription factors, might also be reversible. Prior to this report, a deacetylase responsible for CheY deacetylation has not been fully investigated (Barak *et al*., 2004; 2006). CobB is known to be a NAD^+^-dependent deacetylase and its eukaryotic orthologues have been shown to regulate many biological processes. Using Western blotting with an anti-acetyl lysine antibody we have shown that AcCheY is deacetylated by CobB. Data from LC-MS/MS analysis showed that lysine residues 91 and 109 in AcCheY are acetylated in the peptide K(91).KENIIAAAQAGASGYVVK(109)PFTAATLEEK.L and no lysine residues were found to be acetylated in the same peptide obtained from D-CheY (Fig. 2). This provides sufficient evidence to support that AcCheY is a substrate of CobB. In addition, previous reports showed six lysine residues 91, 92, 109, 119, 122 and 126 are the main acetylation sites (Barak *et al*., 2004). The differences between the previous and the current results may be due to the following reasons: (i) we used 67% acetonitrile containing 2.5% trifluoroacetic acid (TFA) for extracting the peptides in comparison with 5% formic acid; thus the peptide extraction may not be complete, (ii) the acetylation of lysine residues 92 and 122 of AcCheY may prevent its cleavage by trypsin, and (iii) because the acetyl (C-term) was not considered as variable modification, the acetylation of lysine 119 was not found.

### CheY acetylation affects its interaction with FliM

Although many previous observations (Wolfe *et al*., 1988; Barak *et al*., 1998) suggested that CheY acetylation is involved in chemotaxis, the mechanism is still obscure. Structural and functional studies of CheY have revealed that the acetylation sites of CheY are clustered at the C-terminus and appear to be involved in the binding of CheY to FliM (Lee *et al*., 2001; Dyer and Dahlquist, 2006). In 1998, Ramakrishnan *et al*. measured AcCheY–FliM binding using a protein cross-linking assay, but found the ability of AcCheY to bind to FliM *in vitro* was indistinguishable from that of CheY (Ramakrishnan *et al*., 1998). They favour the possibility that acetylation of CheY affects a post-FliM-binding step. However, the sensitivity of the protein cross-linking method used was low. In fact, SPR analysis, a sensitive technique for measuring molecular interactions, clearly demonstrates that the acetylation of CheY reduces its binding to FliM. (Besides, because the batch of AcCheY is probably heterogeneous and consists of both acetylated and non-acetylated CheY molecules, it is reasonable that the binding values for AcCheY are average values of the at least two populations in this batch. This suggests that the actual *K*_D_ of AcCheY binding to FliM is even higher.) This observation may be the consequence of one or both of the following hypothetical possibilities. (i) The acetyl groups on CheY may affect the structure of the protein. It has been reported that Lys109 forms a hydrogen bond through its ε-amine with the carboxyl group of Asp57, the phosphorylation site (Volz and Matsumura, 1991). If the ε-amine is acetylated, then hydrogen bond will not be able to form and thus protein structure may be changed. (ii) Based on the BeF_3_^-^–CheY–FliM_16_ complex structure (pdb id 1F4V), the interactions between the activated CheY and the N-terminal FliM peptides are mediated with a combination of three types of non-covalent bonding: hydrophobic interactions, hydrogen bonds and salt bridges (Lee *et al*., 2001). Among these interactions, the side-chains of K119 and K122 of CheY form stable salt bridges with the side-chains of D12 and N16 of FliM respectively (Fig. S7). Additionally, the salt bridge between K122 and N16 is replaced by a hydrogen bond in the unphosphorylated CheY–FliM_16_ complex (pdb id 2B1J) (Dyer and Dahlquist, 2006). Interestingly, both K119 and K122 are among the six identified acetylation sites of CheY (Barak *et al*., 2004). Acetylation on the ε-amine groups of K119 and K122 would neutralize these two residues, and result in breaking the two bonds between CheY and FliM, which obviously would destabilize the overall inter-molecule interaction. This notion is consistent with the observation that AcCheY had a weaker binding affinity with FliM than CheY.

### Co-regulation of CheY acetylation and phosphorylation *in vivo*

Earlier studies demonstrated that the contributions of CheA and CheZ to the phosphorylation level of CheY appear to be important (Mayover *et al*., 1999). In 2004, Barak and Eisenbach found that CheA strongly inhibits the acetylation of CheY and that CheZ has the opposite effect (Barak and Eisenbach, 2004). In addition, the presence of Acs enhances the phosphorylation level of CheY. Even though this result suggests that CheY acetylation and phosphorylation may be linked, there is a lack of direct evidence for their co-regulation *in vivo*. Interestingly, we observed that the double deletion mutant Δ(*cheA*) Δ(*cheZ*) showed enhanced responses to repellents, but this phenotype could be reversed by further deletion of *cobB*. This result may be interpreted to mean that the phosphorylation level of CheY changed in the absence of CheA and CheZ, and that this change was affected by the deletion of *cobB*. These findings strongly suggest that CobB is involved in the *E. coli* chemotaxis via CheY deacetylation, and that the acetylation and phosphorylation of CheY affect each other *in vivo*. Further experiments will be necessary to unravel the precise mechanism by which these two modifications are co-regulated.

### Potential Role of Acs in acetylating CheY *in vivo*

Although it has been reported that the acetylation of CheY is carried out by Acs (Barak *et al*., 1992) or by autoacetylation (Barak *et al*., 2006), the role of Acs on acetylating CheY *in vivo* is uncertain. Based on our observations that the Δ*acs* mutant shows similar chemotactic responses to the wild-type strain W3110, three hypothetical situations may be considered: (i) in spite of the fact that Acs can probably transfer acetyl groups to CheY *in vitro* (Barak *et al*., 2004), the effect is minor *in vivo*, (ii) CheY undergoes autoacetylation with acetyl-CoA as the acetyl group (Barak *et al*., 2006), and the acetylation level is mainly the outcome of autoacetylation *in vivo* (Yan *et al*., 2008), and (iii) an unknown acetyltransferase is required for the acetylation of CheY. In any case, Acs has little or no effect on the acetylation of CheY *in vivo*.

### CobB regulates *E. coli* chemotaxis by deacetylating CheY

Our observations show: (i) AcCheY is a substrate of CobB, (ii) acetylation of CheY affects the binding affinity between CheY and FliM, (iii) *E. coli*Δ*cobB* has intact flagellum, and (iv) *E. coli*Δ*cobB* had reduced chemotactic responses in the presence of both attractants and repellents. Taken together, these data strongly suggest CobB regulates *E. coli* chemotaxis by deacetylating CheY. And this proposition is further supported by the fact that deletion of *cobB* reverses the effect of a double knockout of *cheA* and *cheZ*. Since *in vitro* experiments have shown that CobB can catalyse the deacetylation of both Acs and CheY, the effect of CobB on chemotaxis may arise from its ability to deacetylate Acs or CheY or both. To distinguish these three possibilities, Δ*acs* and Δ(*cobB*) Δ(*acs*) mutants were used as controls. Chemotactic assays showed that the Δ*acs* mutant was indistinguishable from W3110 and the Δ(*cobB*) Δ(*acs*) mutant had similar phenotype to Δ*cobB* mutant (Fig. 5 and Fig. S5). Therefore, we suggest that CobB works through something other than Acs, perhaps via CheY. In comparison with W3110 and the Δ*acs* mutant, the higher level of CheY acetylation in the Δ*cobB* mutant (Fig. 6) is consistent with the possibility that loss of CobB activity decreases the rate of CheY deacetylation.

## Experimental procedures

### Materials

AcCoA, NAD^+^, NAM, synthetic l-leucine, d-galactose and NiSO_4_ were purchased from Sigma. All chemicals used were of the highest purity available. Anti-acetyl-lysine antibody was obtained from Cell Signaling Technology, and alkaline phosphatase-conjugated goat anti-rabbit antibody was from Sigma. Capillary pipettes (Drummond ‘Microcaps’, 1 µl capacity) were from Fisher Scientific.

### Bacterial strains

*Escherichia coli* strains constructed in this work were derived from strain W3110, a K-12 derivative that is wild type for chemotaxis. All the strains and plasmids used are listed in Table 1. Strains AJW613 and AJW803 were generous gifts of A.J. Wolfe (Loyola University Chicago, Maywood, IL). The gene knockout mutants were constructed with the λ Red recombination system as previously described (Datsenko and Wanner, 2000). The mutants were verified by junction PCR and subsequent sequencing using primers that anneal to the genomic region outside the recombination locus. The double or triple gene knockout mutants were generated using the same procedures, except that the kanamycin resistance gene of the single-gene knockout mutant was first eliminated using the helper plasmid pCP20. All primers used are listed in Table S3.

**Table 1:** Bacterial Strains and plasmids used in this study.

Strain or plasmid	Relevant genotype/phenotype	Reference or source
*E. coli* strains		
W3110	Wild type for chemotaxis; F- λ- *IN(rrnD-rrnE)1 rph-1*	Laboratory stock
RL001	W3110 Δ*cobB*::Km	This study
RL002	W3110 Δ*acs*::Km	This study
RL0012	W3110 Δ*cobB*Δ*acs*::Km	This study
RL003	W3110 Δ*cheA*Δ*cheZ*::Km	This study
RL0031	W3110 Δ*cheA*Δ*cobB*Δ*cheZ*::Km	This study
RL0032	W3110 Δ*cheA*Δ*acs*Δ*cheZ*::Km	This study
AJW613	Wild type for chemotaxis; Δ*lacX74 thi-1 thr-1*(Am) *leuB6 metF159*(Am) *rpsL136*λ*lacY*	A.J. Wolfe
AJW803	AJW613 Δ*acs*::Km-1	Kumari *et al*. (1995)
DH5α	Host for plasmid propagation	Laboratory stock
BL21(DE3)	Host for protein expression	Laboratory stock
AD494(DE3)	Host for protein expression, Km^R^	Laboratory stock
Plasmids		
pKD46	Red recombinase expression plasmids, Ap^R^	Yale CGSC
pKD4	Template plasmids containing a kanamycin resistance gene flanked by FRT sites, Ap^R^, Km^R^	Yale CGSC
pCP20	FLP helper plasmid, Ap^R^, Cm^R^	Yale CGSC
pET20b-*cheY*	CheY-His expression plasmids, Ap^R^	This study
pTricHis2C-*acs*	Acs-His expression plasmids, Ap^R^	This study
pET32a-*cobB*	His-CobB expression plasmids, Ap^R^	This study
pET28a-*fliM*	His-FliM expression plasmids, Km^R^	This study

### Expression and purification of recombinant proteins

CobB, Acs, CheY and FliM encoding genes from *E. coli* W3110 genome were cloned into bacterial expression vectors (Table 1). Transformed *E. coli* AD494 (λDE3)/pET32a-*cobB*, *E. coli* DH5α/pTrcHis2C-*acs*, *E. coli* BL21 (λDE3)/pET20b-*cheY* and *E. coli* BL21 (λDE3)/pET28a-*fliM* were induced with isopropyl-β-d-thiogalactopyranoside (IPTG), lysed and purified by nickel affinity chromatography (detailed primer sequence, expression and purification are described in *Supporting information*).

### *In vitro* deacetylation assays

To obtain acetylated CheY, we incubated CheY-His with acetyl-CoA and 50 mM Tris-HCl buffer (pH 8.0) for 20 h at 35°C, then separated AcCheY from the low-molecular-mass components by ultrafiltration (Barak *et al*., 2006). The acetylation level of AcCheY was determined by Western blotting and then mass spectrometry. Deacetylation of AcAcs and AcCheY by His-CobB was performed in 50 mM Tris-HCl buffer (pH 8.0) in the presence or absence of NAD^+^ (1 mM), and in the presence or absence of NAM (10 mM). Specific protein concentrations and reaction times are indicated in figure legends.

### Preparation of anti-CheY antibodies

A polyclonal antibody against CheY was raised by immunization of rabbits with purified CheY protein. CheY antibodies were purified from rabbit serum by affinity chromatography using protein A beads (Beyotime Institute of Biotechnology, Beijing, China) with glycine-HCl as elutant, dialysed against phosphate-buffered saline. The titre of the purified antibody was determined by ELISA.

### Analysis of CheY acetylation level *in vivo*

Strains W3110, RL001 and RL002 were grown to OD_590_ = 0.4–0.5 in Vogel-Bonner minimal medium supplemented with 30 mM acetate (Vogel and Bonner, 1956). Cells were harvested and lysed as described (Yan *et al*., 2008). Equal amounts (30 µg of total protein) of the cell-free extracts were subjected to SDS-PAGE. Three gels were used: one was stained with Coomassie blue, and the other two were used for Western blotting with CheY antibody and an anti-acetyl-lysine antibody.

### Western blot analysis

The samples were separated by SDS-PAGE (12–15% acrylamide) and then transferred to PVDF membranes using a Bio-Rad SD device (Bio-Rad Laboratories) (20–30 min at 15 V). The membrane was blocked overnight at 4°C in 1× TBST (Tris buffered saline plus 0.1% Tween-20) containing 5% NFDM (non-fat dry milk). Primary rabbit anti-CheY (1:5000) or acetylated-lysine (1:1000) polyclonal antibodies were diluted in TBST/1% NFDM and incubated at 37°C for 2 h. The blot was washed with TBST and incubated with 1:5000 dilution of alkaline phosphatase-conjugated goat anti-rabbit antibody for 1 h at 37°C, then detected according to the manufacturer's instructions.

### LC-MS/MS analysis

Prepared digested peptides were analysed with a Finnigan Surveyor HPLC system coupled online with a LTQ-Orbitrap mass spectrometer (Thermo Electron, San Jose, CA) equipped with a nanospray source. Briefly, the peptide mixtures were loaded onto a C18 column (100 µm i.d., 10 cm long, 5 µm resin from Michrom Bioresources, Auburn, CA) using an autosampler. Peptides were eluted with a 0–35% gradient (Buffer A, 0.1% formic acid and 5% ACN; Buffer B, 0.1% formic acid and 95% ACN) over 80 min and detected online in LTQ-Orbitrap mass spectrometer using a data-dependent TOP10 method (Haas *et al*., 2006) (detailed protein digestion, protein and peptide identification are described in *Supporting information*).

### SPR analysis

Interactions between FliM and CheY, FliM and AcCheY were analysed using BIAcore 3000 (BIAcore AB, Uppsala, Sweden) at 25°C. Approximately 1120 RU His-FliM was immobilized on CM5 sensor chip with an amine-coupling protocol of the BIAcore manual. Before sample measurement, the sensor chip was equilibrated with running buffer at a rate of 40 µl min^−1^. The running buffer contained 10 mM HEPES (pH 7.4), 200 mM NaCl, 3 mM EDTA and 0.05% (v/v) P20. Samples were injected at different concentrations at a flow rate of 40 µl min^−1^ for 2 min. After 3–4 min dissociation phase, bound protein was removed with 30 s wash with 10 mM NaOH. No specific binding to a blank flow cell was subtracted to obtain corrected sensorgrams. Equilibrium and kinetic constants were calculated by a global fit to 1:1 Langmuir binding model (BIA evaluation 4.1 software).

### Chemotaxis assays

Swarm assays were carried out on TB semi-solid agar plates [0.2% agar containing kanamycin (50 µg ml^−1^) when needed] as previously described (Adler, 1966). The migration rate was measured as described previously (Wolfe and Berg, 1989). Chemical-in-plug assays were also carried out as described for both attractants and repellents (Tso and Adler, 1974). Briefly, strains were grown in TB and washed twice in motility buffer (10 mM potassium phosphate buffer, pH 7.0; 100 µM EDTA). Bacteria were then homogeneously distributed in semi-solid agar (0.3%), and the stimulus-containing plug (2% agar) was placed at the centre of the plate before solidification of the agar. A modified drop assay was also performed, in which the stimulus (5 µl) was added directly to the centre of the bacteria-containing plate after solidification of the agar (Barak and Eisenbach, 2001). A modified capillary assay for positive chemotaxis was carried out as described (Han and Cooney, 1993). Capillaries containing the attractant were immersed in a suspension of bacteria and incubated for 1 h at 35°C. The number of bacteria in each capillary was determined by inoculating its content on a TB agar plate. If bacteria accumulated in the attractant-contained capillary to a greater extent than in the attractant-free capillary, attraction was considered to have taken place. Capillary assays for negative chemotaxis (chemical-in-pond) were carried out as described (Tso and Adler, 1974).
