# Supplementary material for: CobB regulates Escherichia coli chemotaxis by deacetylating the response regulator CheY
Source: Mol Microbiol. 2010 Apr 13;76(5):1162–74. doi: 10.1111/j.1365-2958.2010.07125.x (PMC2883070; doi:10.1111/j.1365-2958.2010.07125.x)
Supplement: Supplementary file 1 [file mmi0076-1162-SD1.pdf]

Supplementary Experimental procedures

Protein overexpression and purification

CobB, CheY and FliM were over-expressed and purified by the same procedure. *E. coli* AD494 (λDE3)/pET32a-*cobB* was induced by final concentrations of 1 mM IPTG at OD_{600} = 0.6 for 4 h at 25 °C. *E. coli* BL21 (λDE3)/pET20b-*cheY* and *E. coli* BL21 (λDE3)/pET28a-*fliM* were induced by final concentrations of 0.4 mM IPTG at OD_{600} = 0.5 for 7 h at 18 °C. Cells were harvested by centrifugation, resuspended in Binding Buffer [20 mM Tris–HCl (pH 7.9), 500 mM NaCl and 5 mM imidazole], and then disrupted using an Ultrasonic Cell Disruptor (VCX750, SONICS, US). The disrupted suspension was centrifuged and the supernatant was added to nickel chelate column (Amersham Bioscience, USA) pre-equilibrated with Binding Buffer. The column was washed initially with Washing Buffer [20 mM Tris–HCl (pH 7.9), 500 mM NaCl and 60 mM imidazole] and the histidine-tagged protein was eluted with Elution Buffer [20 mM Tris–HCl (pH 7.9), 500 mM NaCl and 200 mM imidazole]. According to the purity in the SDS–PAGE, the peak fractions were pooled, and concentrated by ultrafiltration with Storing Buffer [50 mM Tris–HCl (pH 7.9), 20% glycerol and 5 mM dithiothreitol]. The proteins were centrifuged at 20 000 g for 15 min and the supernatant was stored at –20 °C or –80 °C in aliquots. Protein purity was estimated to be > 90% on the basis of SDS-PAGE. The concentration of protein was measured by the bicinchoninic acid protein assay kit (Beyotime Biotechnology, Beijing, China) according to the manufacturer's protocol. The over-expression and purification of Acs were performed as described above, except that strain *E. coli*

DH5α/pTrcHis2C-*acs* was used. To achieve acetylated Acs, 50 mM NAM was added simultaneously with IPTG during the course of protein expression (12 h, 25 °C) as previously described (Schwer *et al.*, 2006). For an unknown reason, this method did not work for expressing acetylated CheY.

Protein digestion

Gel lanes to be analyzed were excised from SDS-PAGE gels by razor blade and divided into 1 mm^3 pieces. Each section was washed in water and completely destained using 50% acetonitrile in 25 mM ammonium bicarbonate. A reduction step was performed by addition of 100 μl of 10 mM DTT at 57°C for 1 hr. The proteins were alkylated by adding 100 μl of 55 mM iodoacetamide and allowed to react in the dark at 20 °C for 30 min. Gel sections were first washed in water, then acetonitrile, and finally dried by SpeedVac (Thermo Fisher Scientific, Waltham, MA) for 30 min. Digestion was carried out using 20 μg/ml sequencing grade modified trypsin (Promega, USA) in 50 mM ammonium bicarbonate. Sufficient trypsin solution was added to swell the gel pieces, which were kept at 4 °C for 45 min and then incubated at 37 °C overnight. The supernatants were transferred into a 200 μl microcentrifuge tube and the gels were extracted once with extraction buffer (67% acetonitrile containing 2.5% trifluoroacetic acid). The peptide extract and the supernatant of the gel slice were combined and then completely dried in a SpeedVac centrifuge.

Protein and peptide identification

All the raw data files were processed using BioWorks 3.3.1 (Thermo Finnigan, San Jose, CA) and the derived peak list was searched using the MASCOT search engine

(Matrix Science, London, UK) against AC_000091.fasta database(ftp://ftp.ncbi.nih.gov/genomes/Bacteria/Escherichia_coli_K_12_substr_W3110/). The following search criteria were employed: full tryptic specificity was required; two missed cleavages were allowed; Carbamidomethylation was set as fixed modification, whereas Oxidation (M), Acetyl (K), and Acetyl (N-term) were considered as variable modifications. Initial mass deviation of precursor ion and fragment ions were allowed up to 10 ppm and 0.6 Da, respectively. The top protein of the identification result is chemotaxis protein in each sample.

Electron microscopy

Strains W3110 and the mutant derivatives were grown on the TB medium until the exponential phase. Cells were then harvested and washed gently with 10 mM phosphate buffer (pH 7.0), allowed to stick to a carbon-coated grid for 3 to 4 min, and then negatively stained with 2% phosphotungstic acid for 7 to 8 min (Armstrong *et al.*, 1967). The shape and number of flagella per cell were determined with the aid of a transmission electron microscope.

References

Armstrong, J. B., J. Adler & M. M. Dahl, (1967) Nonchemotactic mutants of Escherichia coli. *Journal of bacteriology* **93**: 390-398.

Schwer, B., J. Bunkenborg, R. O. Verdin, J. S. Andersen & E. Verdin, (2006) Reversible lysine acetylation controls the activity of the mitochondrial enzyme acetyl-CoA synthetase 2. *Proceedings of the National Academy of Sciences of the United States of America* **103**: 10224-10229.

Table S1. Mass spectrometry confirms Lysine-91 is acetylated on AcCheY.

#	b	b^{++}	Seq.	y	y^{++}	#
1	171.1128	86.0600	K			28
2	300.1554	150.5813	E	2749.4407	1375.2240	27
3	414.1983	207.6028	N	2620.3981	1310.7027	26
4	527.2824	264.1448	I	2506.3552	1253.6812	25
5	640.3664	320.6869	I	2393.2711	1197.1392	24
6	711.4036	356.2054	A	2280.1870	1140.5972	23
7	782.4407	391.7240	A	2209.1499	1105.0786	22
8	853.4778	427.2425	A	2138.1128	1069.5600	21
9	981.5364	491.2718	Q	2067.0757	1034.0415	20
10	1052.5735	526.7904	A	1939.0171	970.0122	19
11	1109.5949	555.3011	G	1867.9800	934.4936	18
12	1180.6321	590.8197	A	1810.9585	905.9829	17
13	1267.6641	634.3357	S	1739.9214	870.4644	16
14	1324.6855	662.8464	G	1652.8894	826.9483	15
15	1487.7489	744.3781	Y	1595.8679	798.4376	14
16	1586.8173	793.9123	V	1432.8046	716.9059	13
17	1685.8857	843.4465	V	1333.7362	667.3717	12
18	1813.9807	907.4940	K	1234.6678	617.8375	11
19	1911.0334	956.0204	P	1106.5728	553.7900	10
20	2058.1018	1029.5546	F	1009.5201	505.2637	9
21	2159.1495	1080.0784	T	862.4516	431.7295	8
22	2230.1866	1115.5970	A	761.4040	381.2056	7
23	2301.2238	1151.1155	A	690.3668	345.6871	6
24	2402.2714	1201.6394	T	619.3297	310.1685	5
25	2515.3555	1258.1814	L	518.2281	259.6447	4
26	2644.3981	1322.7027	E	405.1980	203.1026	3
27	2773.4407	1387.2240	E	276.1554	138.5813	2
28			K	147.1128	74.0600	1

Table S2. Mass spectrometry confirms Lysine-109 is acetylated on AcCheY.

#	b	b^{++}	Seq.	y	y^{++}	#
1	129.1022	65.0548	K			28
2	258.1448	129.5761	E	2791.4512	1396.2293	27
3	372.1878	186.5975	N	2662.4087	1331.7080	26
4	485.2718	243.1395	I	2548.3657	1274.6865	25
5	598.3559	299.6816	I	2435.2817	1218.1445	24
6	669.3930	335.2001	A	2322.1976	1161.6024	23
7	740.4301	370.7187	A	2251.1605	1126.0839	22
8	811.4612	406.2373	A	2180.1234	1090.5653	21
9	939.5258	470.2665	Q	2109.0863	1055.0468	20
10	1010.5629	505.7851	A	1981.0277	991.0175	19
11	1067.5844	534.2958	G	1909.9906	955.4989	18
12	1138.6215	569.8144	A	1852.9691	926.9882	17
13	1225.6535	613.3304	S	1781.9320	891.4696	16
14	1282.6535	641.8411	G	1694.9000	847.9536	15
15	1445.7383	723.3728	Y	1637.8785	819.4429	14
16	1544.8067	772.9070	V	1474.8152	737.9112	13
17	1643.8751	822.4412	V	1375.7468	688.3770	12
18	1813.9807	907.4940	K	1276.6783	638.8428	11
19	1911.0334	956.0204	P	1106.5728	553.7900	10
20	2058.1018	1029.5546	F	1009.5201	505.2637	9
21	2159.1495	1080.0784	T	862.4516	431.7295	8
22	2230.1866	1115.5970	A	761.4040	381.2056	7
23	2301.2238	1151.1155	A	690.3668	345.6871	6
24	2402.2714	1201.6394	T	619.3297	310.1685	5
25	2515.3555	1258.1814	L	518.2821	259.6447	4
26	2644.3981	1322.7027	E	405.1980	203.1026	3
27	2773.4407	1387.2240	E	276.1554	138.5813	2
28			K	147.1128	74.0600	1

Table S3. Primers used in this study.

Primer	Sequence (5' to 3') *
cobB deletion	
cobB-1s	<u>ATGCTGTCGCGTCGGGGTCATCGGTTAAGTCGTTTTCGTAAA AATAAACGC</u>ATATGAATATCCTCCTTAG
cobB-2a	<u>TCAGGCAATGCTTCCCGCTTTTAATCCCTTCAGCAACTTTTCAACAAACT</u>TGTAGGCTGGAGCTGCTTCG
acs deletion	
acs-1s	<u>ATGAGCCAAATTCACAAACACACCATTCCTGCCAACATCGCAGACCGTTGC</u>ATATGAATATCCTCCTTAG
acs-2a	<u>TTACGATGGCATCGCGATAGCCTGCTTCTCTTCAAGCAGCTTCTC GACTA</u>TGTAGGCTGGAGCTGCTTCG
cheA deletion	
cheA-1s	<u>GTGAGCATGGATATAAGCGATTTTTATCAGACATTTTTTGATGAAGCGGAC</u>ATATGAATATCCTCCTTAG
cheA-2a	<u>TCAGGCGGCGGTGTTCGCCATACGTTGTTCGCGGTTTATCGCCTGCAAGG</u>TGTAGGCTGGAGCTGCTTCG
cheZ deletion	
cheZ-1s	<u>ATGATGCAACCATCAATCAAACCTGCTGACGAGCATTCAGCTGGCGATATC</u>ATATGAATATCCTCCTTAG
cheZ-2a	<u>TCAAAATCCAAGACTATCCAACAAATCGTCCACCTGATCCTGACTGGCTA</u>TGTAGGCTGGAGCTGCTTCG
Mutant Verification	
cobB	s: ATCTCTTACCTGTAGCTCGTGTTCCG; a: AAAAGTGCGCGTGTATTATTCCG
acs	s: CCCCTATGTGTAACAAATAACCA; a: TATCAGGCCTACAAACCGTTAC
cheA	s: AGCCTGCTGGTACTGAACAA; a: CTCTTCATCACCAAGGGTAAATA
cheZ	s: GTTGCAGGCAGGCGGTTATGGA; a: CGCCCACCAGCAAAATCAGCAG
Protein Expression	
cobB	s: TAAA**GAATTC**TGCTGTCGCGTCGGGGT; a: TAAT**CTCGAG**TCAGGCAATGCTTCCCGC
acs	s: TATA**GGATCC**GATGAGCCAAATTCACAAACACA; a: GCAT**GAATTC**CGATGGCATCGCGATAGC
cheY	s: CGCG**CATATG**GCGGATAAAGAACTTAAATT; a: ATAT**CTCGAG**CATGCCCAGTTTCTCAAAGAT
fliM	s: CGTG**CATATG**GGCGATAGTATTCTTTCTCA; a: TATA**CTCGAG**TTATTTGGGCTGTTCCTCGTTC

*Sequences which are homologous to the deleted gene are underlined, and boldface type indicates restriction sites.

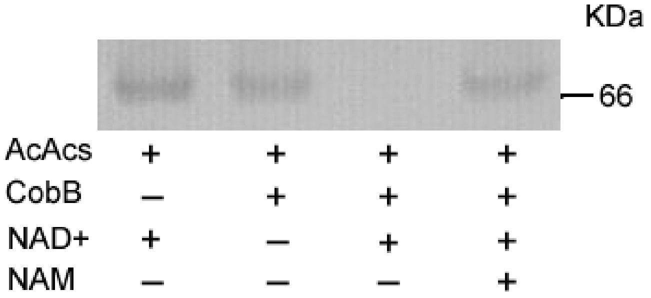

Fig. S1. CobB deacetylates AcAcs *in vitro*. The acetylation levels of AcAcs proteins were determined by Western blot using specific anti-acetyl lysine antibody.

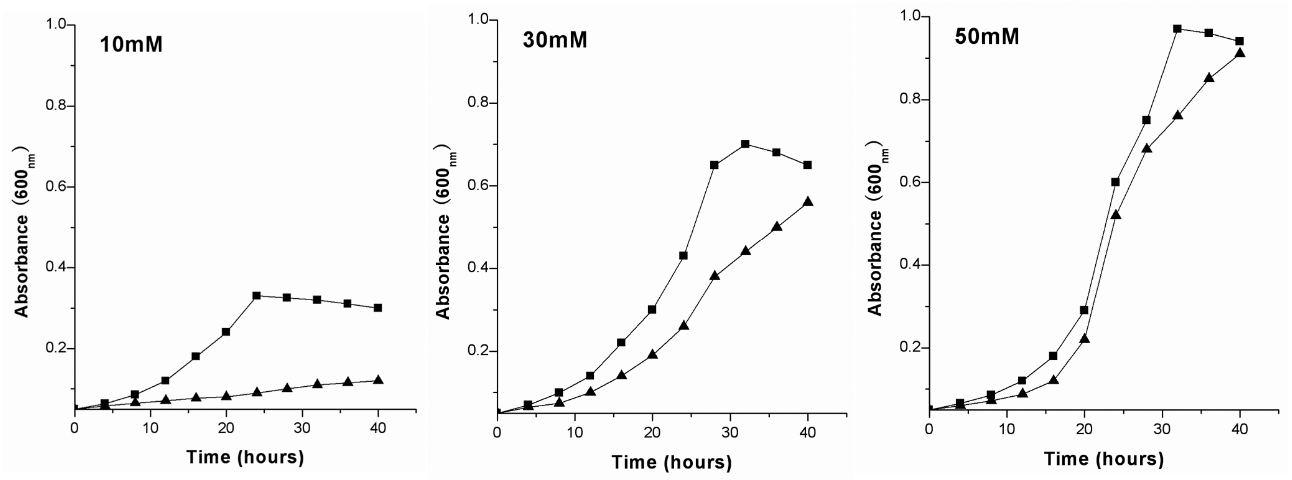

Fig. S2. Growth of W3110 and Δ*cobB* mutant on acetate. The concentration of acetate in the Vogel-Bonner minimal medium was as indicated. The growth behavior of strains W3110 and W3110 Δ*cobB* mutant is shown by squares and triangles, respectively.

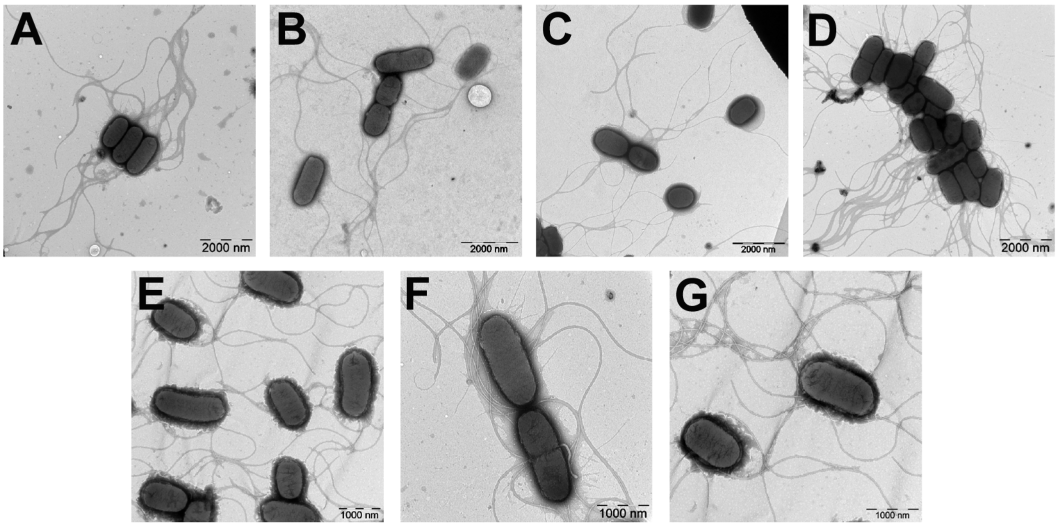

Fig. S3. Electron micrographs of the strains. The bacteria were grown on the TB medium to the exponential phase, washed gently with 10 mM phosphate buffer (pH 7.0), and stained with 2% phosphotungstic acid.

A. Wild type strain W3110. B. W3110 Δ*cobB* mutant. C. W3110 Δ*acs* mutant. D. W3110 Δ(*cobB*) Δ(*acs*) mutant. E. W3110 Δ(*cheA*) Δ(*cheZ*) mutant. F. W3110 Δ(*cheA*) Δ(*cheZ*) Δ(*cobB*) mutant. G. W3110 Δ(*cheA*) Δ(*cheZ*) Δ(*acs*) mutant.

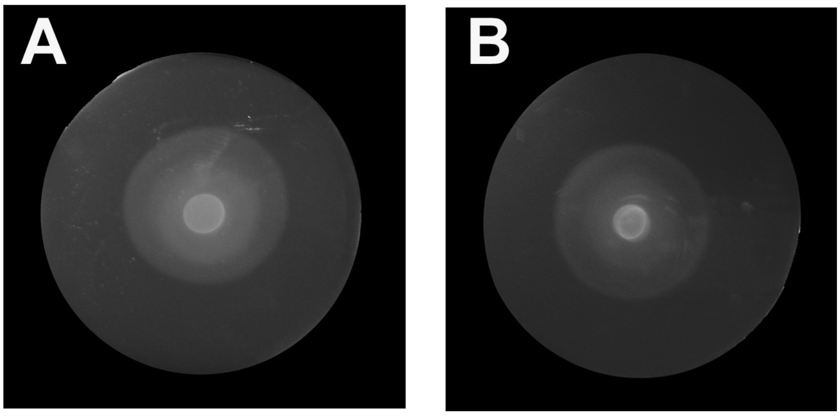

Fig. S4. Swarm assays on TB semisolid plates. The photographs were taken after 4 h of incubation at 35 °C.

A. Swarm ring formation by wild type strain AJW613.

B. Swarm ring formation by Δ*acs* mutant AJW803.

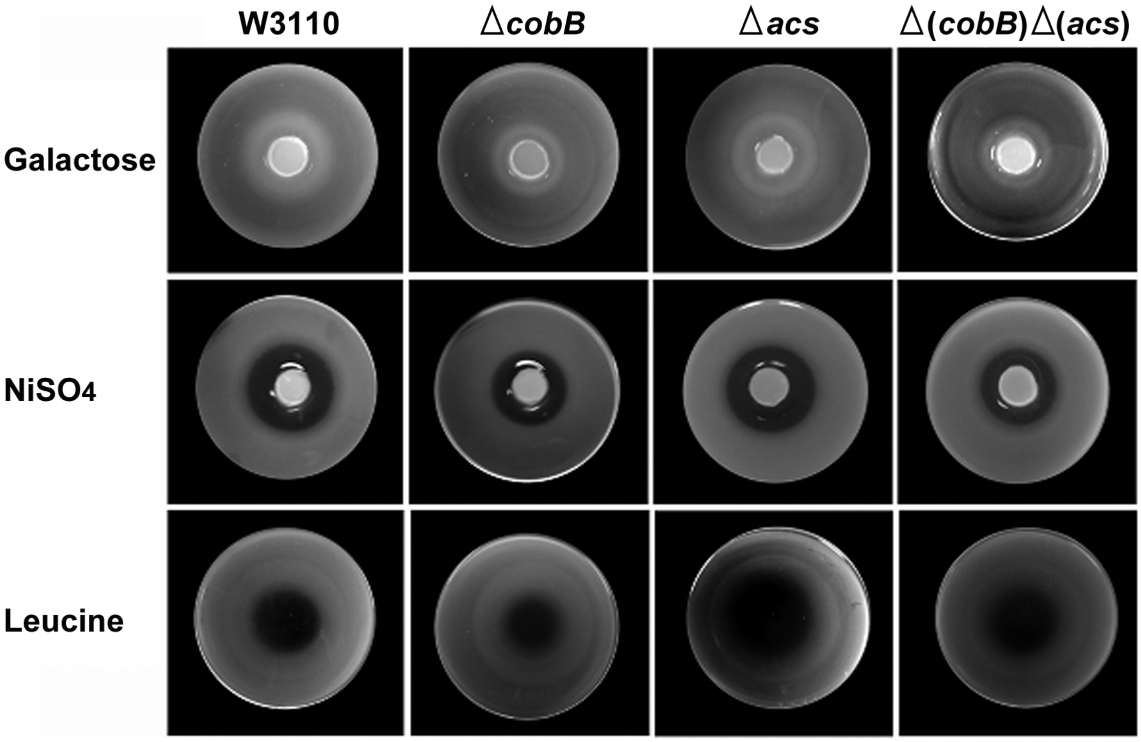

Fig. S5. Plug assays and drop assays of W3110, Δ*cobB* mutant, Δ*acs* mutant and Δ(*cobB*) Δ(*acs*) mutant. The concentrations of galactose plug, $NiSO_4$ plug and leucine drop were 50 mM, 25 mM and 100 mM, respectively. The medium was supplemented with 30 mM acetate. Experiments were replicated four times, and representative results are shown.

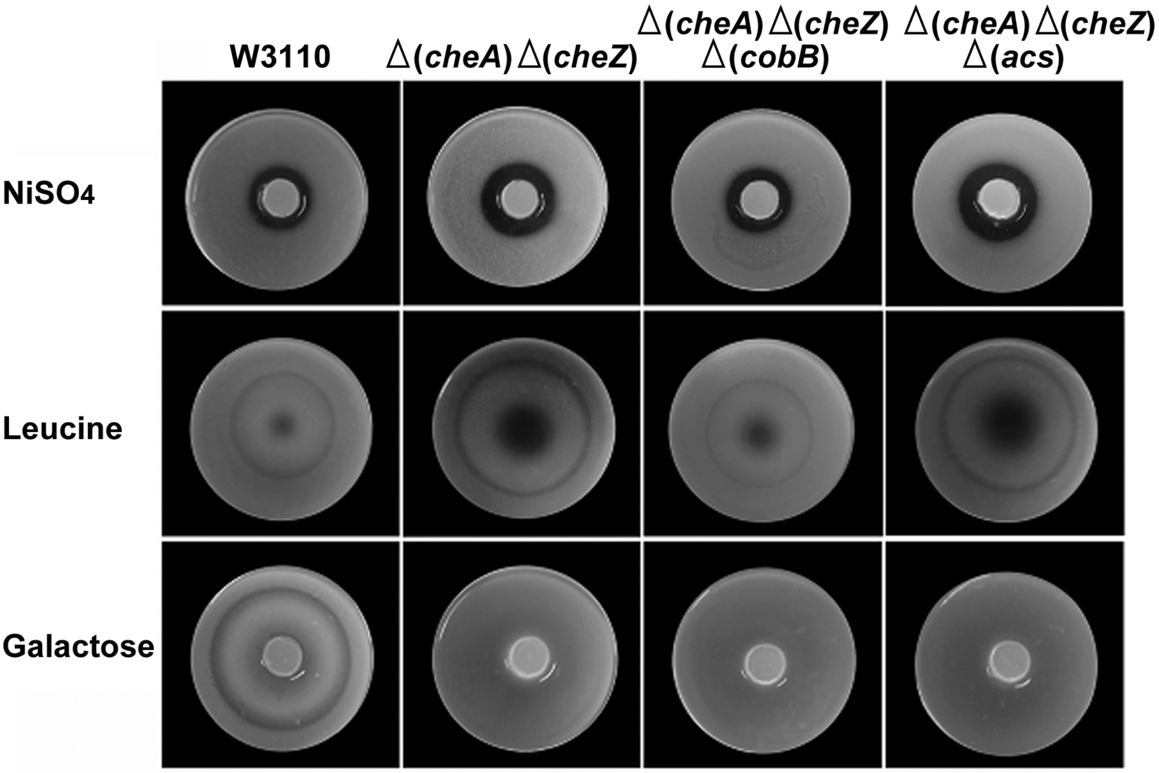

Fig. S6. Plug assays and drop assays of W3110, Δ(*cheA*) Δ(*cheZ*), Δ(*cheA*) Δ(*cheZ*) Δ(*cobB*) and Δ(*cheA*) Δ(*cheZ*) Δ(*acs*). The concentrations of $NiSO_4$ plug, leucine drop and galactose plug were 25 mM, 100 mM and 50 mM, respectively. Experiments were replicated four times, and representative results are shown.

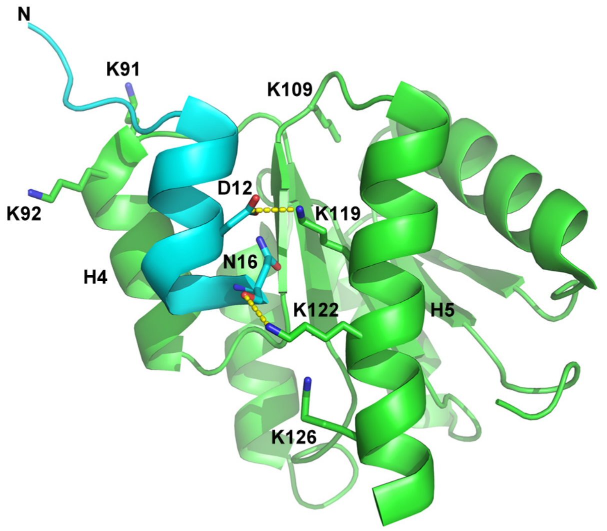

Fig. S7. Cartoon diagram of the structure of BeF_3^--CheY-$FliM_{16}$ complex. $FliM_{16}$ is shown in cyan and CheY is shown in green. Broken yellow lines highlight salt bridges between K119 and D12, K122 and N16.
